# Supplementary material for: Interleukin-1β Drives Disease Progression in Arrhythmogenic Cardiomyopathy
Source: bioRxiv. 2024 Dec 17:2024.12.11.628020. Preprint. [Version 1] doi: 10.1101/2024.12.11.628020 (PMC11702578; doi:10.1101/2024.12.11.628020)
Supplement: Supplement 1 [file media-1.pdf]

List of Supplemental Materials

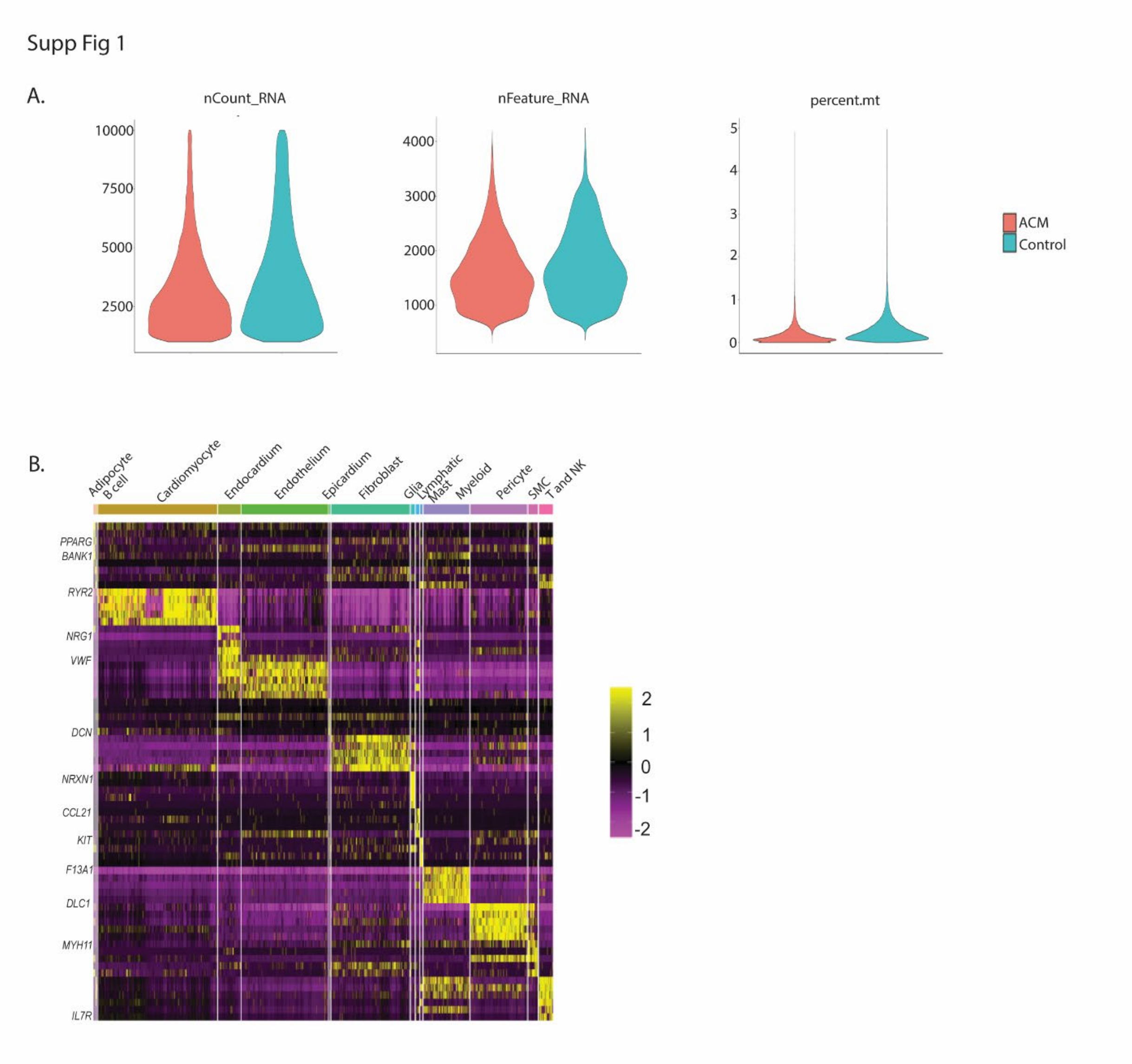

fig S1 QC metrics for human myocardial samples.

(**A**) Major QC cutoffs for single nuclei RNA sequencing data. RNA count: 1000<n<10000, Feature count: 500<n<4000, Mitochondrial percentage: n<5. (**B**) Heatmap displaying major canonical gene expression across different cell types.

Sup Fig 2

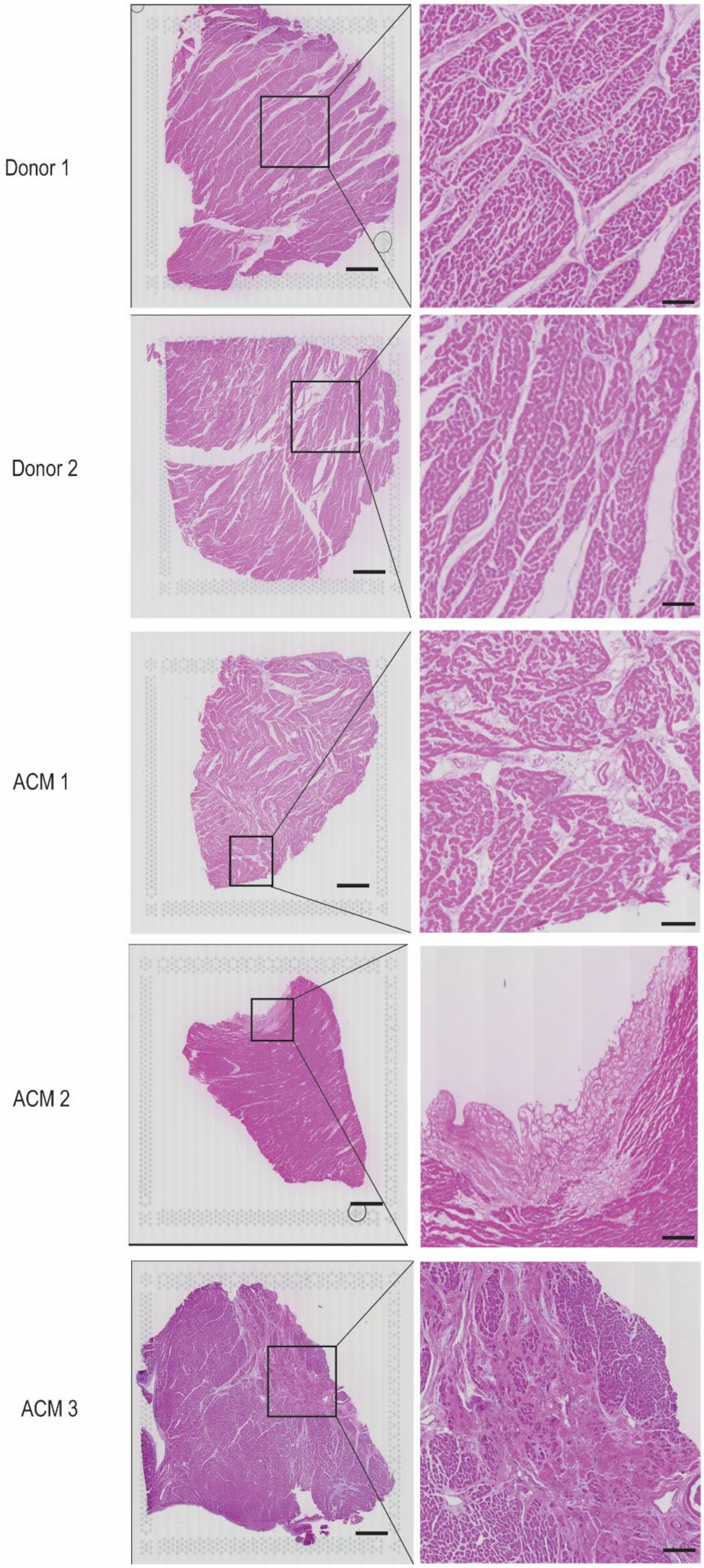

Fig S2 H&E images used for Visium spatial transcriptomics

H&E images for each sample used for spatial transcriptomics. Images to the left were captured on an Axioscan Z7. Images to the right are zoomed insets of areas of healthy myocardium (in donor samples) or areas of ACM lesions (in ACM samples).

Supp Fig 3

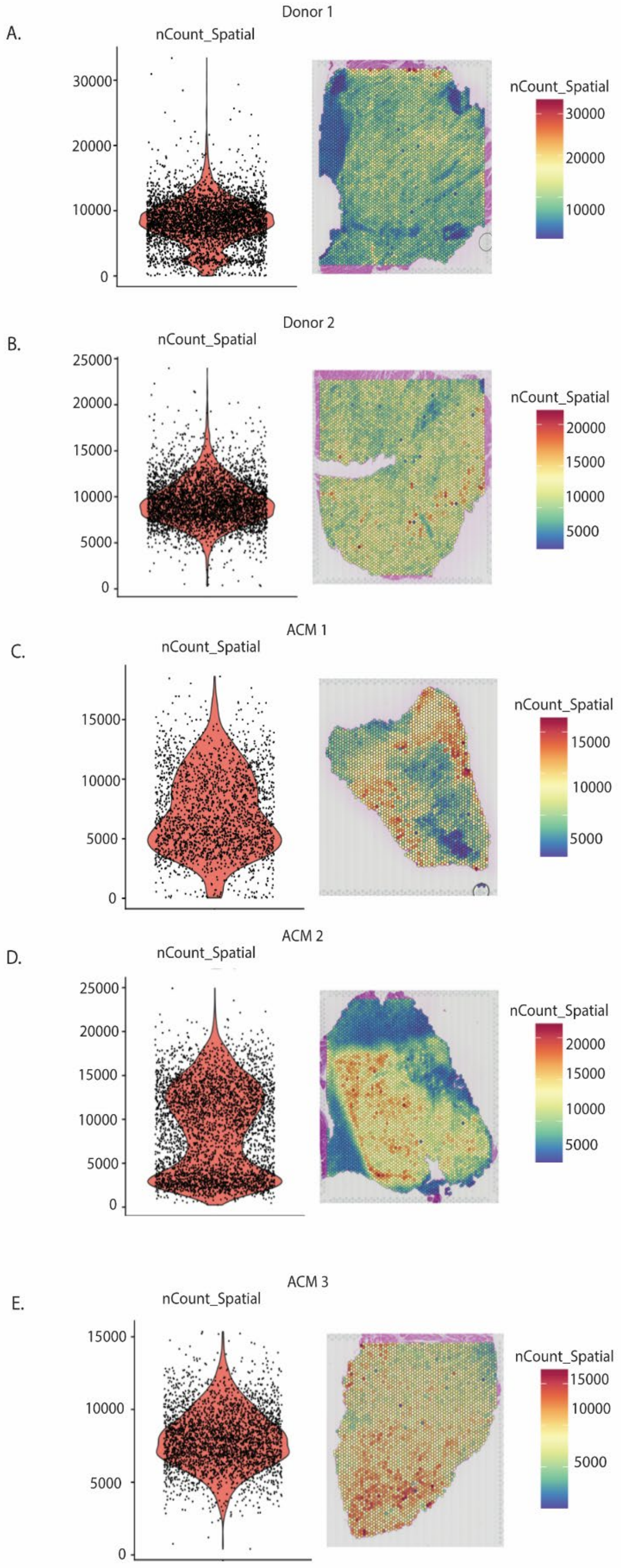

Donor 1
A.
nCount_Spatial
30000
20000
10000
0
nCount_Spatial
30000
20000
10000
Donor 2
B.
nCount_Spatial
25000
20000
15000
10000
5000
0
nCount_Spatial
20000
15000
10000
5000
ACM 1
C.
nCount_Spatial
15000
10000
5000
0
nCount_Spatial
15000
10000
5000
ACM 2
D.
nCount_Spatial
25000
20000
15000
10000
5000
0
nCount_Spatial
20000
15000
10000
5000
ACM 3
E.
nCount_Spatial
15000
10000
5000
0
nCount_Spatial
15000
10000
5000

Fig S3 QC metrics for spatial transcriptomics samples.

Spatial UMI counts for each sample: (**A**) Donor 1, (**B**) Donor 2, (**C**) ACM 1, (**D**) ACM 2, (**E**) ACM 3.

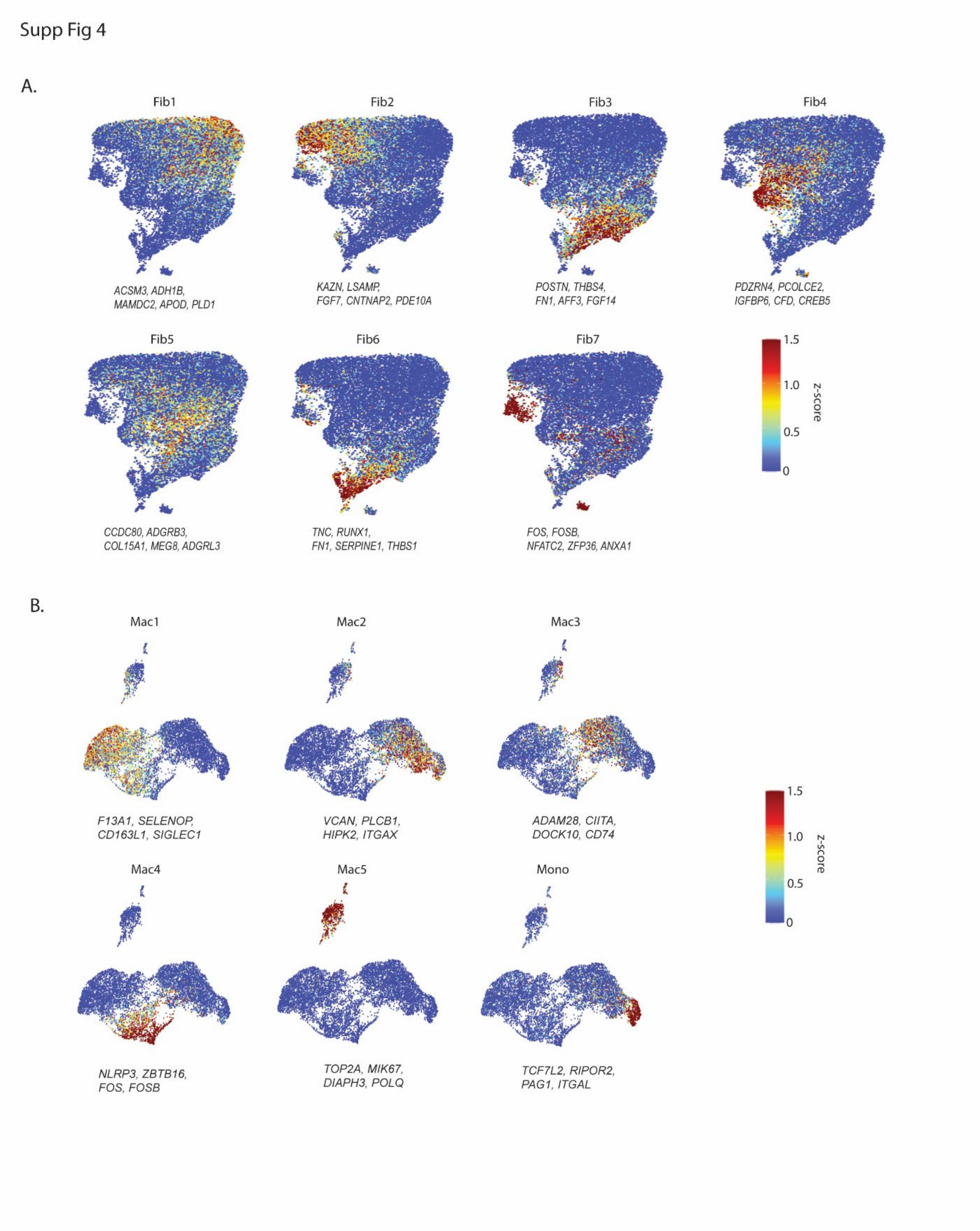

Fig S4 Sub population z-scores for human fibroblasts and myeloid cells.

(**A**) Fibroblast sub populations identified by major gene expression. (**B**) Myeloid sub populations identified by major gene expression.

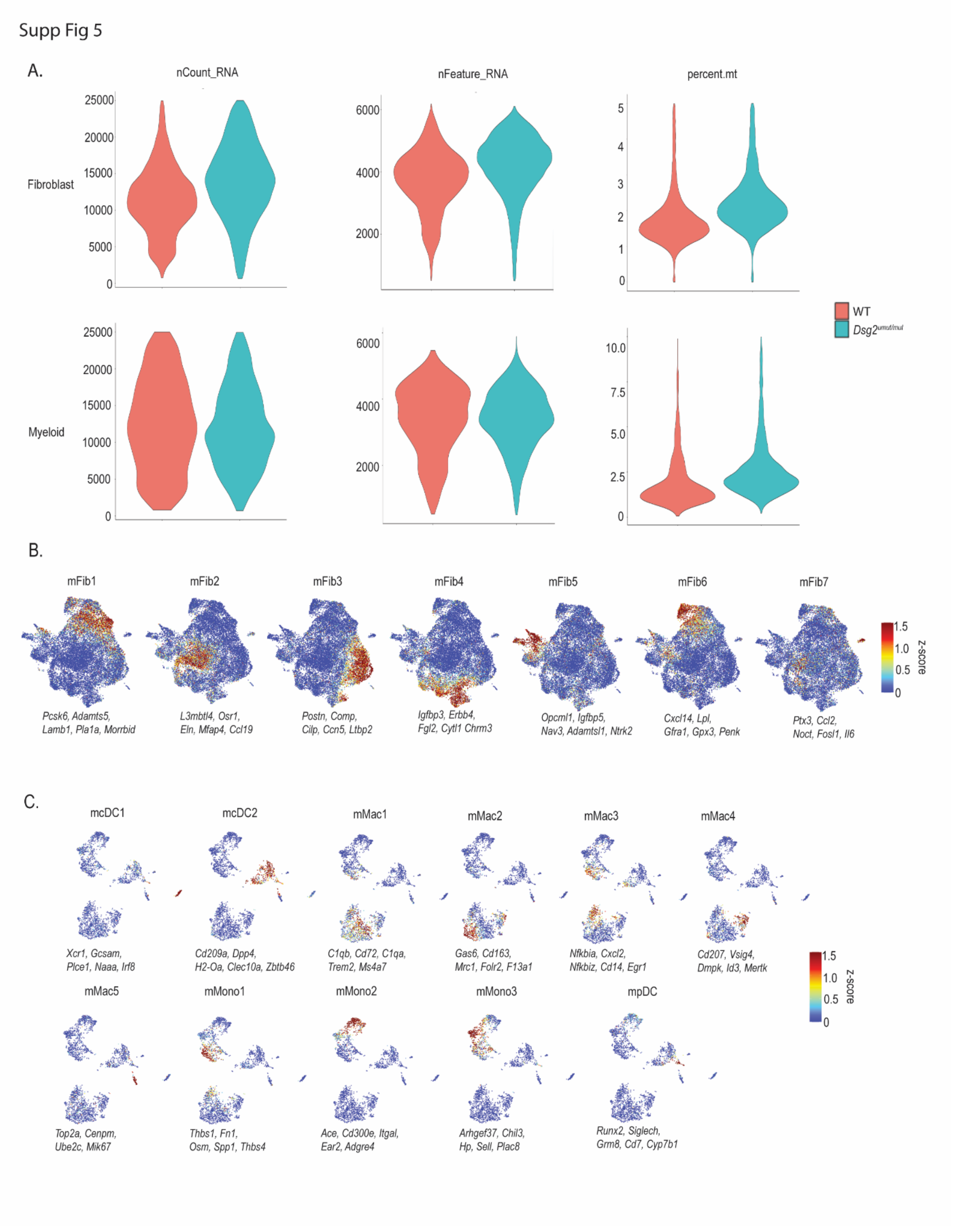

Fig S5 Mouse single cell RNA sequencing QC metrics and major population z-scores.

(**A**) Major QC cutoffs for single cell RNA sequencing data. RNA count: 1000<n<25000, Feature count: 500<n<6000, Mitochondrial percentage: n<5 for fibroblasts and n<10 for myeloid. (**B**) Mouse fibroblast sub populations identified by major gene expression. (**C**) Mouse myeloid sub populations identified by major gene expression.

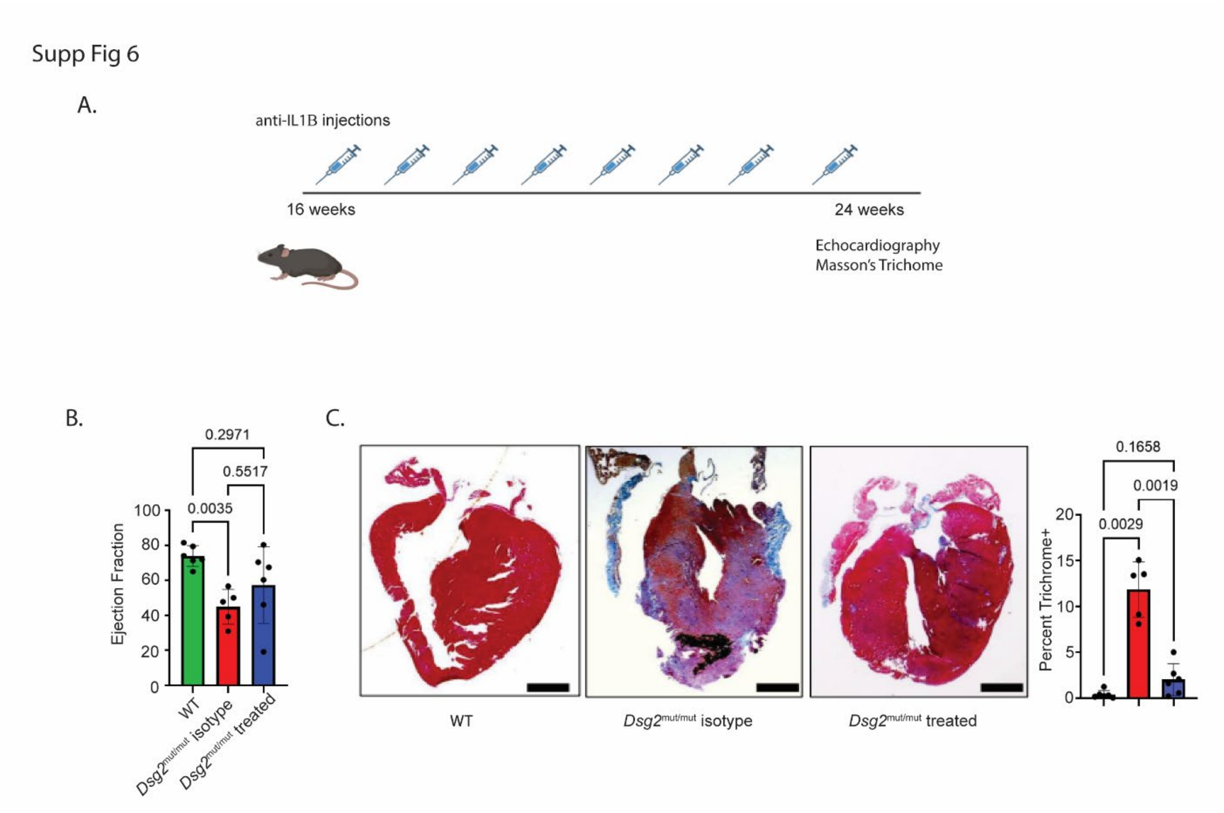

Fig S6 Late IL1β blockade in *Dsg2$^{mut/mut}$* mice

(**A**) Study design outlining treatment schedule of neutralizing IL1β antibody. (**B**) Measurement of ejection fraction (n=6, 5, and 6 for each group respectively). (**C**) Representative Masson's trichrome images from each treatment group and quantification of fibrosis percentage (n=6, 5, and 6 for each group respectively). Brown-Forsythe and Welch ANOVA testing was used for graphs from (**B**) and (**C**).

Supp Fig 7

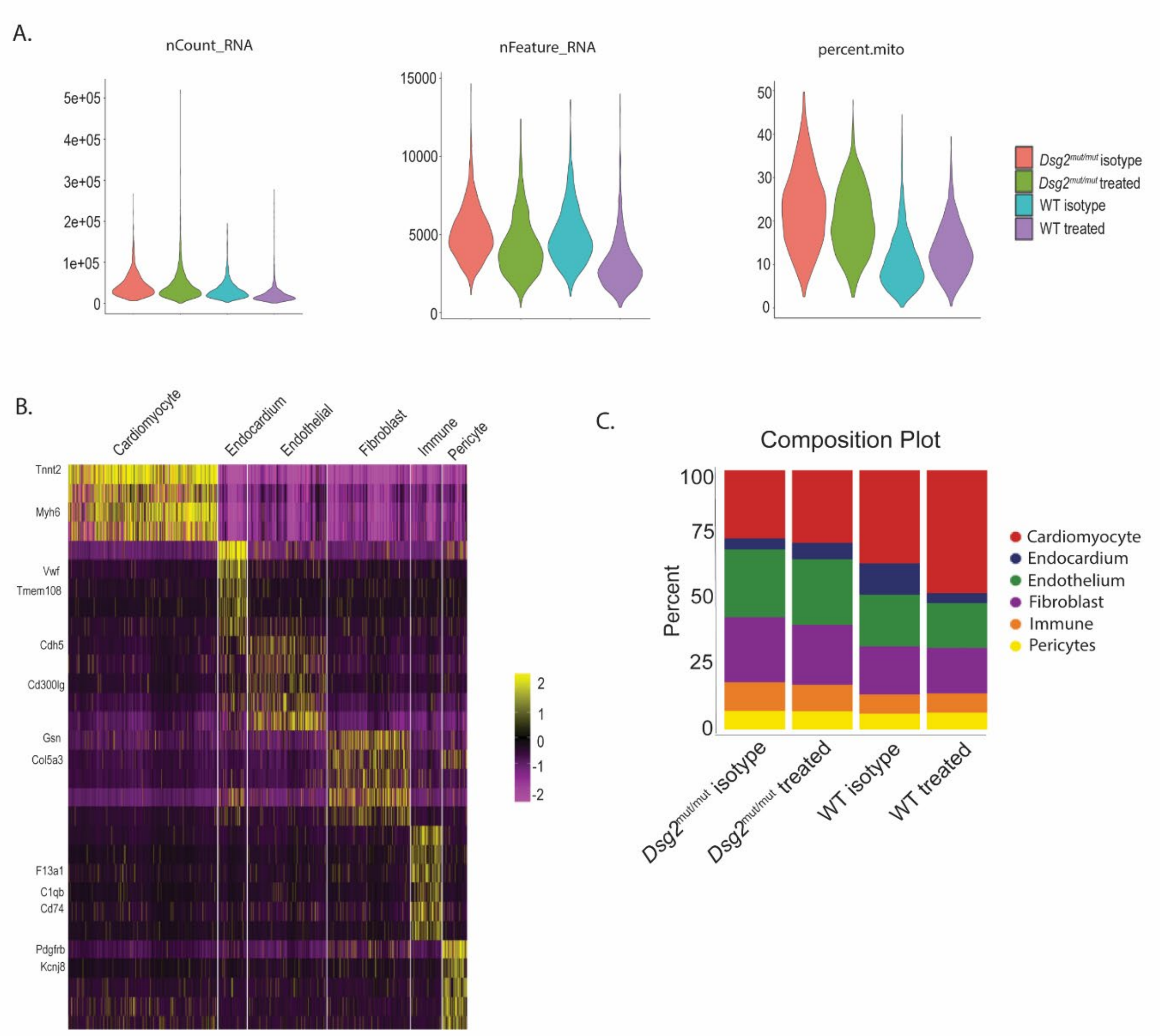

Fig S7 Mouse single nuclei RNA sequencing with iCell8cx system

(**A**) Major QC cutoffs for single cell RNA sequencing data. RNA count: 1000<n<300000, Feature count: 500<n<20000, Mitochondrial percentage: n<30. (**B**) Heatmap displaying major canonical gene expression across different cell types. (**C**) Composition plots comparing major cell populations across treatment groups.

Sample	Condition/Mutation	Gender	Age (years)	DV200	Used for spatial
11-42	Donor control	F	48	39	
11-261	Donor control	F	37	43	X
13-20	Donor control	M	45	37	
13-192	Donor control	F	21	35	
13-198	Donor control	M	11	38	
13-79	Donor control	F	19	42	X
13-96	Donor control	M	28	39	
13-152	Donor control	M	35	30	
P0202	Donor control	F	8	32	
P0172	Donor control	M	7	34	
P0156	Donor control	F	.75	36	
P0159	Donor control	M	1	33	
19-14	*PKP2*	M	46	46	X
20-55	*DSP*	F	13	38	
20-283	*DSP*	M	14	44	X
P0166	*PKP2*	NA	NA	35	
398-100	*PKP2*	F	NA	54	X
T1077	*DSP*	M	61	32	

Table S1 List of human samples used for single nuclei sequencing and spatial transcriptomics

Human Gene Symbol	Mouse ortholog
AFF3	*Aff3*
SMOC2	*Smoc2*
TENM4	*Tenm4*
PDE5A	*Pde5a*
PLCB4	*Plcb4*
IL1RAPL1	*Il1rapl1*
SGIP1	*Sgip1*
PALLD	*Palld*
IGF1	*Igf1*
FAP	*Fap*
DDAH1	*Ddah1*
ASPN	*Aspn*
RUNX2	*Runx2*
MXRA5	*Mxra5*
LTBP2	*Ltbp2*
THBS4	*Thbs4*
MEOX1	*Meox1*
FN1	*Fn1*
POSTN	*Postn*
RUNX1	*Runx1*
CILP	*Cilp*
MEOX2	*Meox2*
NREP	*Nrep*
CCN2	*Ccn2*

Table S2 List of human fibroblast genes (and mouse orthologs) upregulated in ACM used to build gene signature score.

Human Gene Symbol	Mouse ortholog
WASHC2A	*Washc2*
NLRP3	*Nlrp3*
ZBTB16	*Zbtb16*
FOS	*Fos*
CSGALNACT1	*Csgalnact1*
SH3BP5	*Sh3bp5*
LMNA	*Lmna*
EMP1	*Emp1*
GLIS3	*Glis3*
ANXA1	*Anxa1*
ELL2	*Ell2*

FOSB	*Fosb*
ACSL1	*Ascl1*
SRGAP1	*Srgap1*
ARHGEF10L	*Arhgef10l*
NAMPT	*Nampt*
PAPSS2	*Papss2*
TFRC	*Tfrc*
SLC1A3	*Slc1a3*
MAN1A1	*Man1a*
CPM	*Cpm*
SRGN	*Srgn*
IGSF21	*Igsf21*

Table S3 List of human myeloid genes (and mouse orthologs) upregulated in ACM used to build gene signature score.

Fold Change
≤1
1-2
2-4
4-8
8-12
Value

Cytokine	$Dsg2^{mut/mut}$ (Isotpye) vs WT (Isotype)		$Dsg2^{mut/mut}$ (1mg/kg IL-1β) vs WT (Isotype)	
	Fold Change	P-Values	Fold Change	P-Values
CCL6	4-8	P<0.001	1-2	0.874
CCL22	2-4	P<0.05	≤1	0.997
CD14	4-8	P<0.05	1-2	0.630
CD40	8-12	0.108	2-4	P<0.05
CXCL2	8-12	P<0.05	4-8	0.098
CXCL9	2-4	P<0.05	1-2	0.565
IFN-y	8-12	P<0.01	2-4	P<0.05
IGFBP-2	4-8	P<0.05	1-2	0.140
IGFBP-3	2-4	P<0.05	≤1	0.950
IL-1β	8-12	P<0.01	2-4	0.354
IL-3	≤1	0.938	2-4	0.246
MPO	4-8	P<0.05	≤1	0.296
Osteopontin	19	P<0.01	2-4	0.447
Postn	4-8	P<0.05	2-4	0.430

Table S4 Selected list of pro-inflammatory and pro-fibrotic cytokines changed following early IL1β blockade in *Dsg2$^{mut/mut}$* mice.

Full list of genes is available in data supplement.
